# Ruptured Perineum

**Published:** 1880-08

**Authors:** C. C. Frederick

**Affiliations:** House Physician


CASE 2. RUPTURED PERINEUM.—OPERATION REPORTED BY DR. C. C. FREDERICK, HOUSE PHYSICIAN.

Mary Held entered the Buffalo General Hospital, February 6, 1880; aged twenty; occupation domestic; American by birth. She was confined eleven days prior to admission to the Hospital at term, and was attended by a midwife, who allowed the head of the child to rest upon the perineum for twenty-six hours before seeking aid. Dr. Van Peyma was called and delivered her of a dead child with forceps.

Resulting from this long-continued pressure, the perineum and recto-vaginal septum sloughed away for three inches. By March 1st the sloughing surface had healed leaving her entirely unable to control her fecal evacuations. March 18th, Dr. Gay ordered a solution of nitrate of silver, gr. v to the ounce, to be applied to the edges of the wound every two days with camel's hair pencil. The strength of the solution was increased from time to time till it reached gr. xx to the ounce. Dr. Gay operated on April 20th. The parts were denuded of all the cicatricial tissue, which was abundant, and brought together by silver wire sutures—three in the rectal, and four in the perineal portion.

Previous to the operation her bowels had been thoroughly evacuated by a brisk cathartic, and then constipated by opium. A hard rubber tube was placed in the rectum, to allow the escape of flattus, and the patient's knees bound together. She was only allowed a concentrated liquid diet, with pulv. opii. gr. i every six hours, both for the anodyne and constipating effect, and by this means confined her bowels for eleven days.

The parts were irrigated with carbolized water ʒii to oj, twice daily, also gave vaginal douches, and dressed the perineum with a carbolized compress. Catheter passed night and morning. April 30, Dr. Gay removed the sutures.

The perineum is complete, and the recto-vaginal septum restored, with the exception of a small opening scarcely large enough to admit the end of the little finger, just within the verge of the anus. The patient now has complete control of her evacuations.

